# Auer Rod-Like Inclusions in B-Cell Prolymphocytic Leukemia

**DOI:** 10.4274/tjh.galenos.2018.2018.0192

**Published:** 2019-11-18

**Authors:** Yantian Zhao, Juan Lv

**Affiliations:** 1 Beijing Chao-yang Hospital, Capital Medical University, Department of Clinical Laboratory, Beijing, China

**Keywords:** Auer rod-like inclusions, B-cell prolymphocytic leukemia, Lymphocytes

A 76-year-old male patient presented with increasing leukocytes in the past month. Laboratory investigation showed leukocytosis of 30.03x10^9^/L (normal: 3.5-9.5x10^9^/L) with absolute lymphocytosis of 20.7x10^9^/L (normal: 1.1-3.2x10^9^/L), with normal hemoglobin and platelet counts. Review of the peripheral blood smears (Figure 1A) and bone marrow smears (Figure 1B) demonstrated 64% and 74.5% prolymphocytes, respectively, with nucleoli, vacuoles, and Auer rod-like inclusions. The cytoplasmic inclusions were negative for myeloperoxidase by immunohistochemistry. Flow cytometry demonstrated a kappa-restricted CD19 and CD20 immunoreactive B-cell population making up to 67.1% of cells and 93.1% of lymphocytes, with partial expression of sIgM and lacking CD5, CD10, and CD23. No significant expression of CD38 was present. Although Auer rod-like inclusions were seen, there was no evidence of increased immature myeloid cells by flow cytometry or morphology. *IgVH* (FR1-FR3) mutation was not appreciable by molecular biology studies before or during this period. The patient achieved a partial response with chlorambucil treatment.

Auer rod-like inclusions have been reported in B-lineage malignancies like multiple myeloma [1,2]. Electron microscopy revealed these structures to be swollen mitochondria or immunoglobulins [3,4], while classical Auer rods are formed by aggregation and concentration of peroxide granules in myeloid blasts.

## Figures and Tables

**Figure 1:**
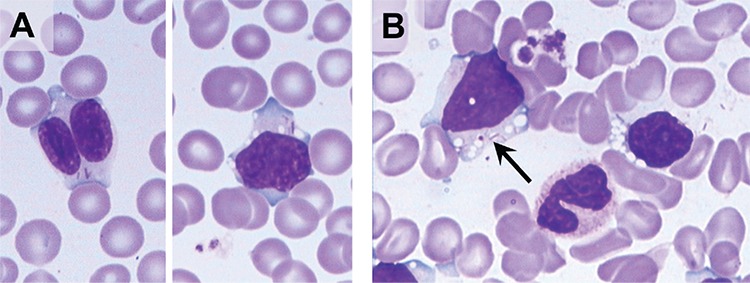
(A) Blood smears and (B) bone marrow smears demonstrating abnormal lymphocytes with Auer rod-like inclusions (1000^x^, Wright-Giemsa stain).
